# Real-space nanoimaging of THz polaritons in the topological insulator Bi_2_Se_3_

**DOI:** 10.1038/s41467-022-28791-x

**Published:** 2022-03-16

**Authors:** Shu Chen, Andrei Bylinkin, Zhengtianye Wang, Martin Schnell, Greeshma Chandan, Peining Li, Alexey Y. Nikitin, Stephanie Law, Rainer Hillenbrand

**Affiliations:** 1 CIC nanoGUNE BRTA, 20018 Donostia - San Sebastián, Spain; 2 Donostia International Physics Center (DIPC), 20018 Donostia - San Sebastián, Spain; 3 Department of Materials Science and Engineering, University of Delaware, Newark, Delaware, 19716 USA; 4 IKERBASQUE, Basque Foundation for Science, 48009 Bilbao, Spain; 5 Wuhan National Laboratory for Optoelectronics & School of Optical and Electronic Information, Huazhong University of Science and Technology, 430074 Wuhan, China; 6 CIC nanoGUNE BRTA and Department of Electricity and Electronics, UPV/EHU, 20018 Donostia-San Sebastián, Spain

**Keywords:** Nanophotonics and plasmonics, Polaritons, Topological insulators

## Abstract

Plasmon polaritons in topological insulators attract attention from a fundamental perspective and for potential THz photonic applications. Although polaritons have been observed by THz far-field spectroscopy on topological insulator microstructures, real-space imaging of propagating THz polaritons has been elusive so far. Here, we show spectroscopic THz near-field images of thin Bi_2_Se_3_ layers (prototypical topological insulators) revealing polaritons with up to 12 times increased momenta as compared to photons of the same energy and decay times of about 0.48 ps, yet short propagation lengths. From the images we determine and analyze the polariton dispersion, showing that the polaritons can be explained by the coupling of THz radiation to various combinations of Dirac and massive carriers at the Bi_2_Se_3_ surfaces, massive bulk carriers and optical phonons. Our work provides critical insights into the nature of THz polaritons in topological insulators and establishes instrumentation and methodology for imaging of THz polaritons.

## Abstract (web summary)

The real-space imaging of propagating THz polaritons, coupled light-matter excitations, in topological insulators has been elusive so far. Here, the authors report spectroscopic THz near-field images of the topological insulator Bi_2_Se_3_ revealing polaritons formed by coupling of THz radiation to optical phonons and various charge carriers.

## Introduction

Plasmon polaritons in metals, doped semiconductors and two-dimensional (2D) materials have wide application potential for field-enhanced spectroscopies, sensing, imaging, and photodetection^1–7^. Recently, topological insulators (TIs) have been attracting large attention as an alternative class of plasmonic materials, as they can support plasmon polaritons that are formed not only by massive but also by Dirac carriers^8,9^. Dirac plasmon polaritons (DPPs) are electromagnetic modes that can be formed when the massless Dirac carriers at the surfaces of a TI collectively couple to electromagnetic radiation^8,10–13^. Due to the 2D nature of these collective excitations, the polariton momentum – and thus the field confinement – is much larger than that of free space photons of the same energy^8,9^, similar to plasmons in 2D materials such as graphene^14,15^. In addition, spin-momentum locking of the electrons in the TI surface states promises additional unique phenomena, such as spin-polarized plasmon waves^16^. For these reasons, DPPs in TIs have attracted significant interest from both a fundamental and applied perspective^17–21^.

DPPs have been reported experimentally by terahertz (THz) far-field spectroscopy of TI microresonator structures^8,9,22^. Due to the unavoidable presence of massive bulk carriers in TI thin films and crystals^9,13,23,24^, however, the analysis and interpretation of the observed THz resonances has been challenging and controversial. The presence of THz bulk phonon polaritons in the prototypical TI Bi_2_Se_3_ further complicates the observation of DPPs^24,25^. Although far-field spectroscopy of TI resonators has provided various fundamental insights, it does not allow for imaging of polariton propagation or mode profiles. Imaging of polaritons – often performed by scattering-type scanning near-field optical microscopy (s-SNOM)^26,27^ - has proven to be of great importance in the infrared spectral range to distinguish between propagating and localized modes in thin layers and resonator structures, for measuring polariton propagation lengths, phase and group velocities, lifetimes and modal field distributions^6,7,14,15,27–30^. However, due to the lack of THz near-field imaging instrumentation offering high spatial and spectral resolution, as well as a large signal-to-noise ratio, the real-space imaging of THz polaritons is still a challenging task^31–33^.

Here, we demonstrate that THz polaritons in TIs can be imaged spectroscopically by s-SNOM employing the tunable monochromatic radiation from a powerful THz gas laser and interferometric detection. Specifically, we performed THz polariton interferometry on epitaxially grown Bi_2_Se_3_ films of different thicknesses $d$. Challenged by the short polariton propagation lengths, we determine the polariton wavevector (and thus dispersion) by complex-valued near-field analysis of our experimental data. Further, using an analytical model, we show that the experimental polariton dispersion can be reproduced when Dirac and massive carriers at the surfaces, massive bulk carriers and optical phonon are taken into account. From propagation length measurements and group velocities determined from the experimental polariton dispersion, we finally determine the decay times of the THz polaritons, amounting to ~0.48 ps and thus being comparable or even better than that of typical plasmon decay times in standard (non-encapsulated) graphene.

## Results

### THz nanoimaging

**Fig. 1:**
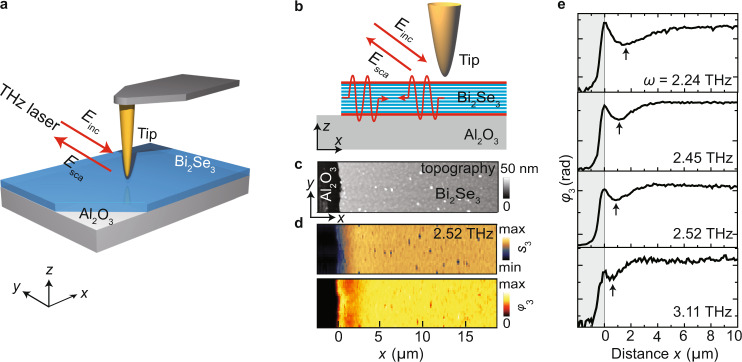
THz s-SNOM imaging polaritons in Bi_2_Se_3_. **a** Schematic of the THz s-SNOM. A parabolic mirror focuses a THz beam onto the apex of an AFM tip. The tip-scattered THz field is collected and recorded interferometrically as function of tip position, simultaneously with topography. **b** Illustration of mapping polaritons (indicated by red sine waves). ${E}_{{{{{{\rm{inc}}}}}}}$ and ${E}_{{{{{{\rm{sca}}}}}}}$ denote the electric field of the incident and tip-scattered radiation. **c** Topography image of a 25-nm-thick Bi_2_Se_3_ film on Al_2_O_3_ and **d** simultaneously recorded amplitude and phase images at a frequency of 2.52 THz. **e** Near-field phase line profiles of the 25 nm thick Bi_2_Se_3_ film at different frequencies, recorded perpendicular to the film edge.

For real space imaging of polaritons in thin Bi_2_Se_3_ films grown by molecular beam epitaxy (Methods section) on sapphire (Al_2_O_3_), we used a THz s-SNOM (based on a commercial setup from Neaspec, Germany; sketched in Fig. 1a; for details see Supplementary Note 1 and Supplementary Fig. 1), where a metallized atomic force microscope (AFM) tip acts as a THz near-field probe. The tip is illuminated with monochromatic THz radiation from a gas laser (SIFIR-50, Coherent Inc., USA), which is focused with a parabolic mirror. Via the lightning rod effect, the tip concentrates the THz radiation into a nanoscale near-field spot at the tip apex^34^. The momenta of the near fields are large enough to launch polaritons in Bi_2_Se_3_. The tip-launched polaritons that propagate to the edge are reflected at the edge and propagate back to the tip (illustrated in Fig. 1b by the red sine waves). Consequently, by recording the tip-scattered field as function of tip position, we map the interference of forward- and backward- propagating polaritons. Collection and detection of the tip-scattered field is done with the same parabolic mirror and a GaAs-based Schottky diode (WR-0.4ZBD, Virgina Diodes Inc. USA). To obtain background-free near-field signals, the tip is oscillated at a frequency Ω (tapping mode) and the detector signal is demodulated at higher harmonics *n* of the oscillation frequency, $n\Omega$. Demodulated near-field amplitude and phase signals, ${s}_{n}$ and ${\varphi }_{n}$, were obtained by synthetic optical holography (SOH), which is based on a Michelson interferometer where the reference mirror (mounted on a delay stage) is translated at a constant velocity along the reference beam path^35,36^. The interferometric detection is key to improve the background suppression and to enable a complex-valued analysis of near-field profiles, which is critical for a reliable measurement of the wavelength of polaritons with short propagation lengths. To increase the signal-to-noise ratio, we used commercial gold tips with a large apex radius^37^ of ~500 nm (Team Nanotec LRCH). They were oscillated at a frequency of about $\Omega$ ≈ 300 kHz with an amplitude of ~200 nm.

Representative THz near-field amplitude and phase images, ${s}_{3}$ and ${\varphi }_{3}$, of a *d* = 25-nm-thick Bi_2_Se_3_ film are shown in Fig. 1d. The phase image reveals a dark fringe on the Bi_2_Se_3_, which is oriented parallel to the film edge (obtained by scratching the film) and resembles s-SNOM images of short-range plasmon and phonon polaritons observed at mid-IR frequencies on graphene and h-BN, respectively^14,38,39^. In contrast, the simultaneously recorded topography image (Fig. 1c) reveals a homogenous thickness of the Bi_2_Se_3_ film, from which we can exclude that the fringe in the THz image is caused by a thickness-dependent dielectric material contrast.

To verify that the dark fringe in Fig. 1d can be attributed to polaritons, we recorded near-field amplitude and phase line profiles perpendicular to the Bi_2_Se_3_ edge, ${s}_{3}(x)$ and ${\varphi }_{3}(x)$, at different THz frequencies $\omega$. The phase profiles ${\varphi }_{3}(x)$ are shown in Fig. 1e. We find that the minimum of the near-field phase signal (marked by an arrow, corresponding to the dark fringe of Fig. 1d) shifts towards the Bi_2_Se_3_ edge with increasing frequency, supporting our assumption that the near-field signal reveals polaritons of several micrometer wavelength. However, the lack of multiple signal oscillators prevents a straightforward measurement of the polariton wavelength.

### Complex-valued analysis of THz line profiles

**Fig. 2:**
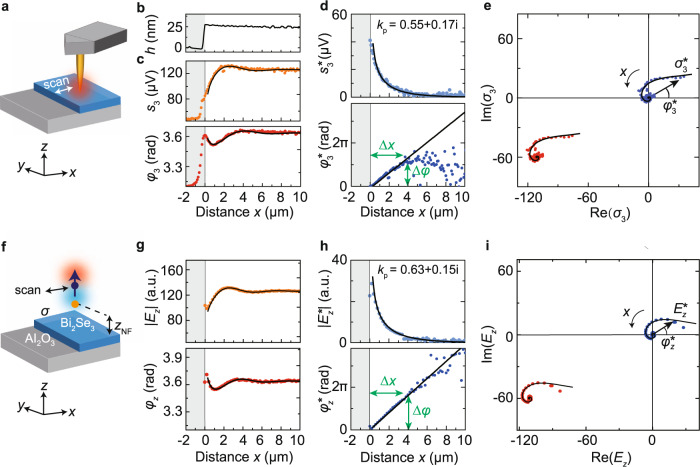
Complex-valued analysis of THz near-field line profiles of a 25-nm-thick Bi_2_Se_3_ film. **a** Sketch of the s-SNOM experiment. **b** Topography line profile, showing the height *h* as measured by AFM. **c** Experimental s-SNOM amplitude and phase line profiles recorded at 2.52 THz. **d** Amplitude and phase line profiles obtained from the data shown in panel c after subtraction of the complex-valued signal offset *C* at large distances $x$. **e** Representation of near-field line profiles in the complex plane. Data corresponding to panel c are shown in red color, data corresponding to panel d are shown in blue color. **c**–**e** The black solid lines show the fitting of the experimental data by a radially and exponentially decaying wave, $A{e}^{i2{k}_{{{{{{\rm{p}}}}}}}x}/\sqrt{2x}+C$, where *C* is a constant complex-valued offset. **f** Simulation of s-SNOM line profiles: A vertically orientated dipole source (mimicking the tip) is located 1.5 μm above a sheet of conductivity *σ* (blue, mimicking the Bi_2_Se_3_ layer) on Al_2_O_3_. The electric field *E*_z_ below the dipole at height *z*_NF_ = 200 nm above the conductivity sheet is calculated and plotted as function of the distance *x* between the dipole and the sheet edge. **g**–**i** Simulated amplitude and phase line profiles analogous to panels **c**–**e**. The conductivity was obtained from Eq. (2) with ${k}_{{{{{{\rm{p}}}}}}}$ = 0.55 + 0.17*i* μm^−1^. For better comparison between the experimental and simulation results, the offset *C* in the simulations (red data) was replaced by the experimental offset and the phases in panels d and h were set to zero a *x* = μm.

To establish a procedure for measuring the polariton wavelengths $\lambda$_p_ and corresponding wavevector ${k}_{{{{{{\rm{p}}}}}}}^{\prime{}}=2\pi /{\lambda}_{{{{{{\rm{p}}}}}}}$, we performed a complex-valued analysis of the THz near-field amplitude and phase line profiles, as illustrated in Fig. 2 with data obtained on a 25-nm-thick Bi_2_Se_3_ film that were recorded at 2.52 THz. We first constructed complex-valued line profiles ${\sigma }_{3}\left(x\right)={s}_{3}{\left(x\right)e}^{i{\varphi }_{3}\left(x\right)}$ and plotted the corresponding trajectories in the complex plane, i.e. as a polar plot where the polar amplitude and phase represent the near-field amplitude ${s}_{3}\left(x\right)\,$and the near-field phase ${\varphi }_{3}(x)$, respectively. We find that the near-field signal describes a spiral (red data in Fig. 2e, based on the line profiles shown in Fig. 2c) around a complex-valued offset *C* that corresponds to the near-field signal at large tip-edge distances $x$. The spiral stems from a harmonic oscillation (describing a circle) whose amplitude decays with increasing $x$, indicating a single propagating mode that is strongly damped. Indeed, after removing the offset (blue data in Fig. 2e), we obtain a monotonically decaying amplitude and a linearly increasing phase signal (Fig. 2d). To verify that the spiral reveals a damped propagating wave, we fitted the complex-valued experimental line profile (red data in Fig. 2e) by

1 $${E}_{{{{{{\rm{p}}}}}}}=A{e}^{i2{k}_{{{{{{\rm{p}}}}}}}x}/\sqrt{2x}+C,$$

which describes the electric field of a back-reflected, radially (i.e. tip-launched) propagating damped wave (black curve in Fig. 2e). The fitting parameters are *A*, ${k}_{{{{{{\rm{p}}}}}}}$ and *C*. ${k}_{{{{{{\rm{p}}}}}}}$is the complex-valued polariton wavevector ${k}_{{{{{{\rm{p}}}}}}}={k}_{{{{{{\rm{p}}}}}}}^{\prime }+i{k}_{{{{{{\rm{p}}}}}}}^{\prime \prime},$ where ${k}_{{{{{{\rm{p}}}}}}}^{ \prime{} }=2\pi /{\lambda }_{{{{{{\rm{p}}}}}}}$ and 1/${k}_{{{{{{\rm{p}}}}}}\,}^{\prime\prime}$is the propagation length. The complex-valued offset $C$ corresponds to the tip-sample near-field interaction in absence of polaritons that are back-reflected from the Bi_2_Se_3_ edge, i.e. when the tip is far away from the edge. *A* is a complex-valued factor. This offset is present in all polariton maps obtained by s-SNOM and described for example in refs. ^40,41^. It can be also seen in our numerical simulations discussed in Fig. 2f–i. After removing the offset *C*, we obtain $A{e}^{-2{k}_{{{{{{\rm{p}}}}}}}^{\prime\prime}x}{e}^{i4\pi x/{\lambda }_{{{{{{\rm{p}}}}}}}}/\sqrt{2x}$, where the term $A{e}^{-2{k}_{{{{{{\rm{p}}}}}}}^{\prime\prime}x}/\sqrt{2x}$ describes a decaying amplitude and the term ${e}^{i4\pi x/{\lambda }_{{{{{{\rm{p}}}}}}}}$ a linearly increasing phase $\varphi \,=\,4\pi x/{\lambda }_{{{{{{\rm{p}}}}}}}$ when the distance *x* to the Bi_2_Se_3_ edge increases. The linear relation between distance $x$ and phase $\varphi$ thus reveals directly the polariton wavelength according to ${\lambda }_{{{{{{\rm{p}}}}}}}\,=\,4\pi {{{{{\rm{\cdot }}}}}}\Delta x/\Delta \varphi$. The fits (black curves in Fig. 2c–e) match well the experimental data, in particular the linear increase of the phase (Fig. 2d) when *C* is removed, which is the key characteristics of a propagating mode. The fit yields a wavevector of ${k}_{{{{{{\rm{p}}}}}}}$ = 0.55 + 0.17*i* μm^−1^, corresponding to a normalized wavevector $q$ = ${k}_{{{{{{\rm{p}}}}}}}/{k}_{0}$ = 10.4 + 3.2*i*, where ${k}_{0}$ is the photon wavevector. Note that for fitting we excluded the first 200 nm from the edge, in order to avoid a potential influence of tip-edge near-field interaction and edge modes (see discussion below). In the Supplementary Figs. 2 and 3 of the Supplementary Note 2 we show all recorded line profiles and fittings reported in this work.

To verify our analysis of the experimental s-SNOM profiles and the determination of the polariton wavevector ${k}_{{{{{{\rm{p}}}}}}}$, we performed well-established numerical model simulations^30,42^. As illustrated in Fig. 2f, the s-SNOM tip is described by a vertically orientated dipole source and the Bi_2_Se_3_ layer by a 2D sheet of an optical conductivity $\sigma$ (blue layer in Fig. 2f). The electric field ${E}_{{{{{{\rm{z}}}}}}}=\left|{E}_{{{{{{\rm{z}}}}}}}\right|{e}^{i{\varphi }_{z}}$ (describing the s-SNOM signal) below the dipole is calculated and plotted as function of the distance *x* between the dipole source and the sheet edge (mimicking the scanning of the tip, Fig. 2g). To obtain the sheet conductivity $\sigma$, we assume that optical polariton modes are probed in our experiment (where the polariton fields normal to the film have opposite sign at the top and bottom surface; for further discussion see below). In this case, $\sigma$ can be obtained from the dispersion relation of polaritons in a 2D sheet within the large momentum approximation^43,44^:

2 $$q(\omega )=\frac{{k}_{p}}{{k}_{0}}=\,i\frac{c}{4\pi }\frac{{\varepsilon }_{{{{{{\rm{sub}}}}}}}+1}{\sigma (\omega )},$$

where *q* is the normalized complex-valued polariton wavevector along the film, $\omega$ the frequency, and ${\varepsilon }_{{{{{{\rm{sub}}}}}}}$ = 10 the permittivity of the Al_2_O_3_ substrate^9,24,45^ (see Supplementary Note 3 and Supplementary Fig. 4). We note that the approximation of the layer by a 2D sheet conductivity is justified, despite Bi_2_Se_3_ being an anisotropic material hosting hyperbolic polaritons, as the layers are much thinner than the polariton wavelength and the damping of the polaritons is rather high (for further details see Supplementary Fig. 5 and Supplementary Notes 3 and 4). For ${k}_{{{{{{\rm{p}}}}}}}$ = 0.55 + 0.17*i* μm^−1^ (according to our analysis of the experimental s-SNOM line profiles in Fig. 2c–e), we obtain the simulated near-field line profiles shown in Fig. 2g–i. An excellent agreement with the experimental s-SNOM line profiles is found. Particularly, complex-valued fitting of the simulated line profiles by a radially decaying wave (according to Eq. (1)) yields a polariton wavevector ${k}_{{{{{{\rm{p}}}}}}}$ = 0.63 + 0.15*i* μm^−1^, which closely matches the value determined from the experimental s-SNOM line profiles. The simulations thus confirm that the experimental s-SNOM line profiles reveal a polariton mode that (i) is launched by the tip, (ii) propagates as a damped wave radially along the Bi_2_Se_3_ film, (iii) reflects at the edge of the film back to the tip, and (iv) is scattered by the tip.

**Fig. 3:**
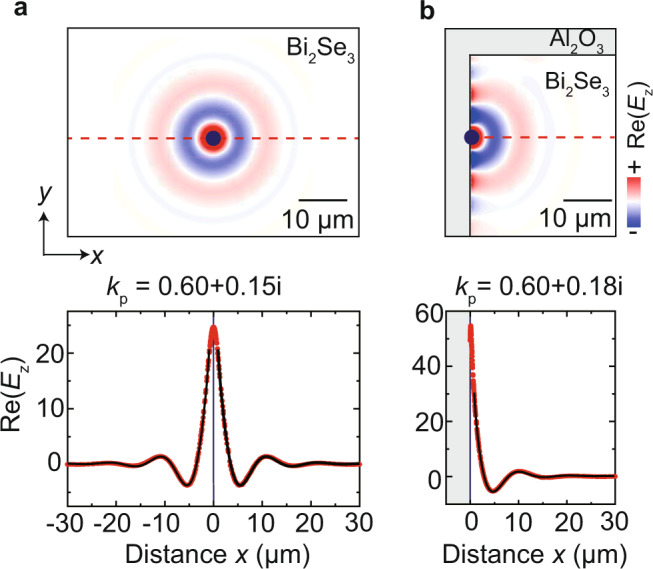
Simulation of the near-field distribution of polaritons launched by a dipole source in a conductivity sheet. The conductivity was obtained from Eq. (2) with ${k}_{{{{{{\rm{p}}}}}}}$ = 0.55 + 0.17*i* μm^−1^. **a** Dipole source is located far away from edges. **b** Dipole source is located at the sheet edge. The lower panels show the electric field along the horizontal red dashed line in upper panel (red data). The black lines of the lower panels show fits according to a radially propagating damped wave, ${{{{{\rm{Re}}}}}}\left[A{e}^{i{k}_{{{{{{\rm{p}}}}}}}x}/\sqrt{x}\right]$. The height of the dipole above the sheet is 1.5 μm and the electric field was calculated in a height of 200 nm.

For a demonstration of our conclusions, we show the electric near-field distribution around the dipole source placed above the conductivity sheet, ${{{{{\rm{Re}}}}}}\left[{E}_{z}\left(x,y\right)\right].$ When the dipole is placed inside the sheet, i.e. far away from any edge, we clearly observe radially propagating wavefronts (Fig. 3a, upper panel). Most important, fitting of the field distribution along the horizontal dashed red line by ${{{{{\rm{Re}}}}}}\left[A{e}^{i{k}_{{{{{{\rm{p}}}}}}}x}/\sqrt{x}\right]$ yields ${k}_{{{{{{\rm{p}}}}}}}$ = 0.60 + 0.15*i* μm^−1^ (Fig. 3a, lower panel), which agrees well with the wavevectors ${k}_{{{{{{\rm{p}}}}}}}$ obtained from the experimental and simulated s-SNOM line profiles. The slight discrepancies between the various wavevectors (<15%) may be attributed to the excitation of an edge mode when the tip comes into close proximity of the sheet edge. The edge mode can be actually recognized in the simulations when the dipole is located at the sheet edge (Fig. 3b, upper panel). Its wavelength is slightly reduced compared to that of the sheet mode, and its fields are strongly confined to the edge, similarly to what has been observed for plasmon and phonon polariton modes in graphene and h-BN flakes^30,46,47^. Most important, since the edge mode propagates exclusively along the edge and its field is strongly confined to the edge, its contribution in the experimental and simulated s-SNOM line profiles perpendicular to the edge (shown in Fig. 2) is minor, when we allow small uncertainties below 15%.

### Analysis of the THz polariton dispersion

**Fig. 4:**
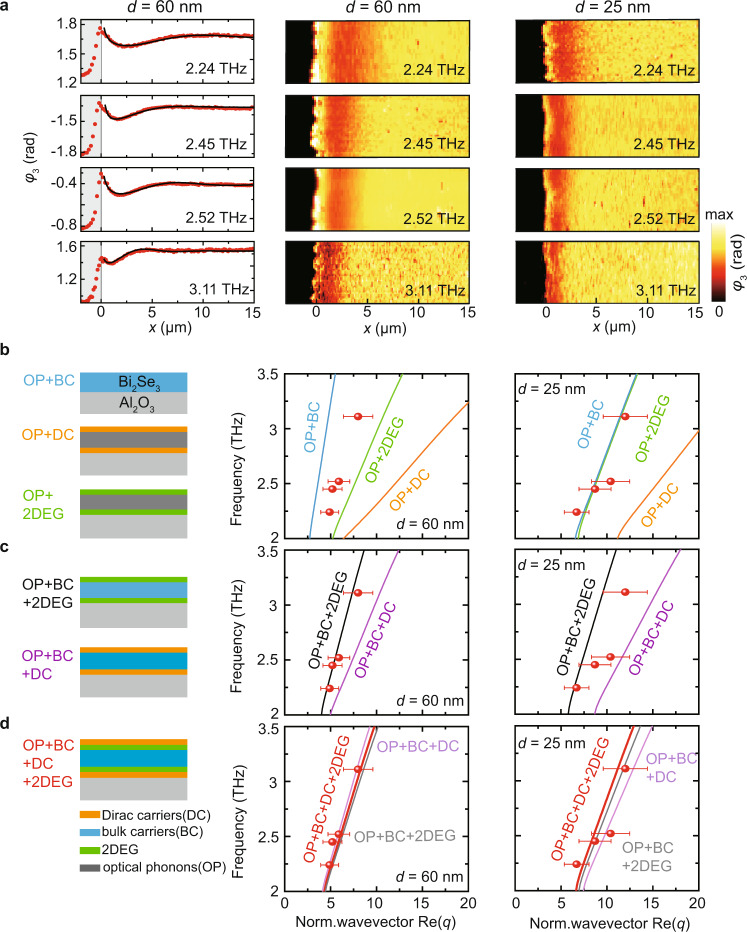
Polariton dispersions in Bi_2_Se_3_ films with 60 nm and 25 nm thickness. **a** Right: near-field phase images at different THz frequencies. Left: line profiles extracted from the images of the 60 nm thick film. Experimental phase line profile are shown in red color. Back lines show fits obtained by complex-valued fitting as demonstrated in Fig. 2. **b**–**d** Red symbols in the diagrams show the polariton dispersions obtained by complex-valued fitting of experimental line profiles as demonstrated in Fig. 2. Solid lines show calculated dispersions based on various conductivity models (described in main text), which are sketched on the left side. We consider various optical conductivity contributions based on optical bulk phonons (OP), massive bulk carriers (BC), Dirac carriers (DC) at both Bi_2_Se_3_ surfaces, and massive two-dimensional electron gases (2DEG) at both Bi_2_Se_3_ surfaces. Error bars indicate a 20% uncertainty of the wavevector, which we estimate conservatively from comparison of experimental and simulated near-field line profiles.

In Fig. 4a we show the phase images of two Bi_2_Se_3_ films of different thicknesses recorded at different frequencies, which were used to determine the polariton dispersions according to the procedure described in Fig. 2 (red symbols in Fig. 4b; $q$ = ${k}_{{{{{{\rm{p}}}}}}}/{k}_{0}$ is the polariton wavevector ${k}_{{{{{{\rm{p}}}}}}}$ normalized to the photon wavevector ${k}_{0}$). As is typical for polaritons, we find that ${{{{{\rm{Re}}}}}}[q]$ increases with increasing frequency $\omega$ and with decreasing film thickness *d*. To understand the physical origin of the polaritons, we compare the experimental results with analytical calculations of the polariton dispersion employing Eq. (2) and various conductivity models describing the Bi_2_Se_3_ film (see Supplementary Note 4, Supplementary Fig. 6 and Supplementary Table 1). For modeling of the conductivity, we performed Hall measurements of Bi_2_Se_3_ films of different thicknesses (see Supplementary Fig. 7 and Supplementary Notes 5 and 6), yielding an effective 2D carrier concentration of about ${n}_{2{{{{{\rm{D}}}}}},{{{{{\rm{Hall}}}}}}}$ = 2.5 × 10^13^ cm^−2^ for layers with a thickness of ~25 nm (Supplementary Fig. 8).

First, we assume that only optical phonons (OP) and Dirac carriers (DC) located on both film surfaces contribute to the conductivity, yielding ${\sigma =\sigma }_{{{{{{\rm{OP}}}}}}+{{{{{\rm{DC}}}}}}}^{{{{{{\rm{model}}}}}}}=\frac{\omega d}{4\pi i}{\varepsilon }_{{{{{{\rm{phonon}}}}}}}+\,2{\sigma }_{{{{{{\rm{Dirac}}}}}}},$ where ${\varepsilon }_{{{{{{\rm{phonon}}}}}}}$ is the bulk dielectric function of Bi_2_Se_3_ including optical phonons. ${\sigma }_{{{{{{\rm{Dirac}}}}}}}$ is the sheet conductivity of one Bi_2_Se_3_ surface (see Supplementary Eq. (8))^11,12,25^, assuming that the sheet carrier concentration at one surface is ${n}_{{{{{{\rm{Dirac}}}}}}}={n}_{2{{{{{\rm{D}}}}}},{{{{{\rm{Hall}}}}}}}/2$ = 1.25 × 10^13^ cm^−2^ (independent of the thickness). The resulting dispersions are shown by the orange curves in Fig. 4b. We find that the calculated wavevectors are significantly larger than the experimental values, from which we conclude that pure Dirac plasmon polaritons coupled to phonon polaritons cannot explain the experimental dispersion. As a second case, we assume that all carriers are bulk carriers (BC), yielding ${\sigma =\sigma }_{{{{{{\rm{OP}}}}}}+{{{{{\rm{BC}}}}}}}^{{{{{{\rm{model}}}}}}}=\frac{\omega d}{4\pi i}({\varepsilon }_{{{{{{\rm{phonon}}}}}}}+{\varepsilon }_{{{{{{\rm{Drude}}}}}}}),$ where ${\varepsilon }_{{{{{{\rm{Drude}}}}}}}$ is the Drude contribution to the bulk dielectric function (see Supplementary Note 4). In this case, we use an effective three-dimensional (3D) concentration of the massive carriers according to ${n}_{{{{{{\rm{bulk}}}}}}}$ = ${n}_{2{{{{{\rm{D}}}}}},{{{{{\rm{Hall}}}}}}}$/25 nm = 1 × 10^19^ cm^−3^. We obtain the dispersions shown by the light blue curves in Fig. 4b. For the 25 nm thick film a reasonable match of the experimental polariton wavevectors is found, however, not for the 60 nm thick film, revealing that polaritons comprising only bulk carriers (BC) and optical phonons (OP) cannot explain the polariton dispersions either. A similar observation is made (green curves in Fig. 4b) when we assume that all carriers stem from a massive 2D electron gas (2DEG) - which is known to exist in TIs due to surface band bending^18,48,49^ - yielding ${\sigma =\sigma }_{{{{{{\rm{OP}}}}}}+2{{{{{\rm{DEG}}}}}}}^{{{{{{\rm{model}}}}}}}=\frac{\omega d}{4\pi i}{\varepsilon }_{{{{{{\rm{phonon}}}}}}}+2{\sigma }_{2{{{{{\rm{DEG}}}}}}}$, where ${\sigma }_{2{{{{{\rm{DEG}}}}}}}$ (see Supplementary Eq. (9))^11,12,49^ is the sheet conductivity of one Bi_2_Se_3_ surface with ${n}_{2{{{{{\rm{DEG}}}}}}}={n}_{2{{{{{\rm{D}}}}}},{{{{{\rm{Hall}}}}}}}/2$ = 1.25 × 10^13^ cm^−2^.

We next assume that both massive bulk carriers and surface carriers (either Dirac or massive 2DEG carriers) contribute to the conductivity,$\,{\sigma =\sigma }_{{{{{{\rm{OP}}}}}}+{{{{{\rm{BC}}}}}}+{{{{{\rm{DC}}}}}}}^{{{{{{\rm{model}}}}}}}=\frac{\omega d}{4\pi i}({\varepsilon }_{{{{{{\rm{phonon}}}}}}}+{\varepsilon }_{{{{{{\rm{Drude}}}}}}})+2{\sigma }_{{{{{{\rm{Dirac}}}}}}}$ and ${\sigma =\sigma }_{{{{{{\rm{OP}}}}}}+{{{{{\rm{BC}}}}}}+2{{{{{\rm{DEG}}}}}}}^{{{{{{\rm{model}}}}}}}=\frac{\omega d}{4\pi i}({\varepsilon }_{{{{{{\rm{phonon}}}}}}}+{\varepsilon }_{{{{{{\rm{Drude}}}}}}})+2{\sigma }_{2{{{{{\rm{DEG}}}}}}}$, respectively. Note that massive bulk carriers in thin films are barely captured by the Hall measurements (due to their supposedly smaller mobility compared to that of the Dirac carriers; see Supplementary Note 6). We thus assign the total Hall-measured 2D concentration (${n}_{2{{{{{\rm{D}}}}}},{{{{{\rm{Hall}}}}}}}$ = 2.5 × 10^13^ cm^−2^) fully to either Dirac or massive 2DEG carriers for both the 25 nm and 60 nm thick film, and consider an additional massive bulk carrier concentration ${n}_{{{{{{\rm{bulk}}}}}}}$. From Hall measurements of thick Bi_2_Se_3_ films we estimate ${n}_{{{{{{\rm{bulk}}}}}}}$ = 2.15 × 10^18^ cm^−3^ (Supplementary Note 6), yielding the bulk Drude contribution ${\varepsilon }_{{{{{{\rm{Drude}}}}}}}$. The calculated dispersions (black and purple lines Fig. 4c, labeled OP+BC+2DEG and OP+BC+DC, respectively) again do not match the experimental results (red symbols).

In the following, we attempt to fit the experimental dispersions by various parameter variations. We first added an additional 2DEG contribution, such that ${\sigma =\sigma }_{{{{{{\rm{OP}}}}}}+{{{{{\rm{BC}}}}}}+{{{{{\rm{DC}}}}}}+2{{{{{\rm{DEG}}}}}}}^{{{{{{\rm{model}}}}}}}=\frac{\omega d}{4\pi i}({\varepsilon }_{{{{{{\rm{phonon}}}}}}}+{\varepsilon }_{{{{{{\rm{Drude}}}}}}})+{2\sigma }_{{{{{{\rm{Dirac}}}}}}}+2{\sigma }_{2{{{{{\rm{DEG}}}}}}}$ (red lines Fig. 4d). Using for each surface the carrier concentrations ${n}_{{{{{{\rm{bulk}}}}}}}$ = 2.15 × 10^18^ cm^−3^ and ${n}_{{{{{{\rm{Dirac}}}}}}}=$ 1.25 × 10^13^ cm^−2^ (from the Hall measurements), we obtain the fitting parameter ${n}_{2{{{{{\rm{DEG}}}}}}}=$ 0.375 × 10^13^ cm^−2^ for each surface. We note that in Hall measurements we cannot separate Dirac and massive carriers directly (Supplementary Note 6) to confirm these carrier concentrations, but they are close to the numbers reported in literature^9,23,50–52^. Interestingly, the experimental dispersions can be also fitted without considering a 2DEG (employing the conductivity model ${\sigma =\sigma }_{{{{{{\rm{OP}}}}}}+{{{{{\rm{BC}}}}}}+{{{{{\rm{DC}}}}}}}^{{{{{{\rm{model}}}}}}}$, light purple line in Fig. 4d). However, we have to assume an increased bulk carrier concentration of ${n}_{{{{{{\rm{bulk}}}}}}}=$ 3.72 × 10^18^ cm^−3^ (fitting parameter; ${n}_{{{{{{\rm{Dirac}}}}}}}\,$as before). Although the required bulk carrier concentration is nearly twice as high as the one estimated from our Hall measurements, it represents a reasonable value reported in literature^9,23,24,49,50^, which may not be fully revealed by Hall measurements (see discussion in Supplementary Note 6). We also fitted the experimental dispersions without considering Dirac carriers employing the conductivity model ${\sigma =\sigma }_{{{{{{\rm{OP}}}}}}+{{{{{\rm{BC}}}}}}+2{{{{{\rm{DEG}}}}}}}^{{{{{{\rm{model}}}}}}}\,$(gray line in Fig. 4d) with ${n}_{{{{{{\rm{bulk}}}}}}}\,$= 2.15 × 10^18^ cm^−3^ and ${n}_{2{{{{{\rm{DEG}}}}}}}$ being the fit parameter. A good matching of the experimental dispersion is achieved for ${n}_{2{{{{{\rm{DEG}}}}}}}=$ 0.95 × 10^13^ cm^−2^ for each surface. However, this value of ${n}_{{{{{{\rm{total}}}}}},2{{{{{\rm{DEG}}}}}}}=$ 1.9 × 10^13^ cm^−2^ is significantly higher than what has been reported in literature^18,49,51^. Altogether, we conclude from our systematic dispersion analysis that an unambiguous clarification of the nature and concentration of carriers forming the polaritons is difficult without additional experiments where the concentrations of the different carriers can be measured separately. However, such measurements are challenging to carry out at room temperature due to thermal smearing, and low-temperature measurements are unlikely to be accurate at room temperature due to thermal excitations. On the other hand, considering that Bi_2_Se_3_ growth is highly reproducible and that Dirac carriers have been verified in samples like ours^53^, these carriers may contribute to the signal.

### Polariton propagation length and lifetime

From the complex-valued fitting of the s-SNOM line profiles we also obtain the propagation length of the polaritons, ${L}={1}/{k}_{{{{{{\rm{p}}}}}}\,}^{\prime\prime}$. For the 25 nm thick film we find $L$ = 6 μm and accordingly the amplitude decay time $\tau =L/{v}_{{{{{{\rm{g}}}}}}}$ = 0.48 ps, which is similar to the decay times measured by far-field extinction spectroscopy of Bi_2_Se_3_ ribbons^9^. The group velocities ${v}_{{{{{{\rm{g}}}}}}}$ were obtained from the polariton dispersion according to ${v}_{{{{{{\rm{g}}}}}}}=d\omega /d{k}_{{{{{{\rm{p}}}}}}\,}^{\prime}$ = 0.042*c*.

Interestingly, the polariton decay time in Bi_2_Se_3_ is comparable or even larger than that of graphene plasmons at infrared frequencies^14,15,28^. On the other hand, the inverse damping ratio of the Bi_2_Se_3_ polaritons, ${\gamma }^{-1}$ = ${k}_{{{{{{\rm{p}}}}}}\,}^{\prime}/{k}_{{{{{{\rm{p}}}}}}\,}^{\prime\prime}$ = 3.2, and their relative propagation length, $L{/\lambda }_{{{{{{\rm{p}}}}}}}=\frac{1}{2\pi \gamma }=$ 0.5, is significantly smaller than that of the infrared graphene plasmons (${\gamma }^{-1}$ = 5)^15^. To understand the small relative propagation length of the Bi_2_Se_3_ polaritons, we express it as a function of the polariton wavevector ${k}_{{{{{{\rm{p}}}}}}}=q\omega /c$, group velocity ${v}_{{{{{{\rm{g}}}}}}}\,$and decay time $\tau$:

3 $$\frac{L}{{\lambda }_{{{{{\rm{p}}}}}}}=\frac{1}{2\pi }{k}_{{{{{{\rm{p}}}}}}\,}^{\prime}{v}_{{{{{{\rm{g}}}}}}}\tau =\frac{1}{2\pi }\frac{\omega }{c}{{{{{{\rm{Re}}}}}}[q]v}_{{{{{{\rm{g}}}}}}}\tau .$$

**Fig. 5:**
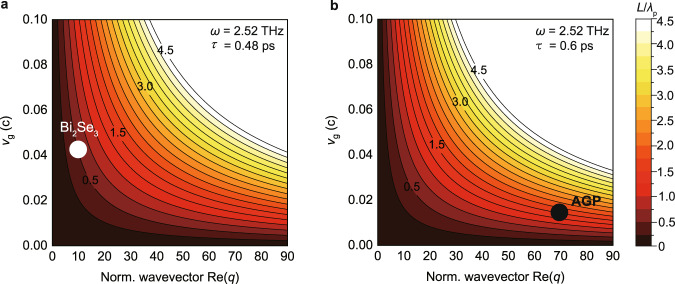
Relative propagation length *L/λ*_p_ of 2.52 THz polaritons as a function of wavevector and group velocity. **a** Polariton amplitude decay time is $\tau$ = 0.48 ps. Black numbers indicate the relative propagation lengths. White symbol shows experimental relative propagation length for THz polaritons in the 25-nm-thick Bi_2_Se_3_. **b** Polariton amplitude decay time is $\tau$ = 0.6 ps. Black numbers indicate the relative propagation length. Black symbol shows the experimental relative propagation length of acoustic graphene plasmon (AGP) of an amplitude decay time of 0.6 ps in high-quality graphene encapsulated in h-BN (data taken from Alonso-Gonzalez et al.^31^).

From Eq. (3) it becomes clear that for a given $\tau$, ${v}_{{{{{{\rm{g}}}}}}}$ and $q$, the relative propagation length decreases with decreasing frequency; that is simply because the temporal oscillation period becomes longer. Since typical THz frequencies are more than one order of magnitude smaller than infrared frequencies, one can expect, generally, that the relative propagation length of THz polaritons in thin layers (including 2D materials) is significantly smaller than that of infrared polaritons. For an illustration, we show in Fig. 5a the calculated relative propagation length $L{/\lambda }_{{{{{{\rm{p}}}}}}}\,$of 2.52 THz polaritons of 0.48 ps amplitude decay time as function of ${{{{{\rm{Re}}}}}}[q]$ and ${v}_{{{{{{\rm{g}}}}}}}$. The white symbol marks the relative propagation length of the THz polaritons observed in the 25-nm-thick Bi_2_Se_3_ film. We find that propagation lengths of more than wavelength, $L{/\lambda }_{{{{{{\rm{p}}}}}}} \, > \, 1$, are possible only for large group velocities (>0.1c) when the normalized polariton wavevector (i.e. polariton confinement) is moderate (${{{{{\rm{Re}}}}}}[q]$ > 10). To achieve $L{/\lambda }_{{{{{{\rm{p}}}}}}} \, > \, 1$ for group velocities below 0.05c, large polariton wavevectors with ${{{{{\rm{Re}}}}}}[q]$ > 20 are required. For comparison, we show in Fig. 5b the relative propagation length for polaritons of 0.6 ps amplitude decay time. Plasmon polaritons of such rather exceptionally large amplitude decay time were observed experimentally in high-quality graphene encapsulated in h-BN (marked by black symbol). Only because of their very large confinement (${{{{{\rm{Re}}}}}}[q]$ = 70; owing to coupling with adjacent metallic gate electrodes), these polaritons possess a large relative propagation length of $L/{\lambda }_{{{{{{\rm{p}}}}}}}=1$.5, although their group velocity is rather small (${v}_{g}$ = 0.014c). Generally, we conclude from Eq. (3) and Fig. 5 that the relative propagation lengths of THz polaritons are generally short, unless THz polaritons of extraordinary long decay times^33^, large wavevectors or large group velocities are studied.

## Discussion

We note that in our experiments we could only observe the optical polariton modes, although s-SNOM in principle can map acoustic polariton modes as well^31^. We explain the absence of acoustic polariton modes (where the sign of the effective surface charges is opposite on both surfaces) by their extremely short wavelengths, which may prevent efficient coupling with the probing tip. Further, the acoustic modes might be strongly overdamped due to the presence of bulk carriers. In the future, sharper tips and TIs of lower bulk carrier concentration which can be grown using a buffer layer technique^54^ and may allow the study of the ultra-confined acoustic modes in real space.

In summary, we demonstrated an instrumentation for s-SNOM that allows for spectroscopic nanoimaging of thin-film polaritons around 2.5 THz, even in case of weak polaritonic image contrasts. We applied it to record real-space images of THz polaritons in the TI Bi_2_Se_3_. Despite the short polariton propagation, we could measure the polariton dispersion and propagation length, owing to complex-valued analysis of near-field line profiles. In the future, the highly specific signature of polaritonic spatial signal oscillations in the complex plane – representing a spiral – could be also applied to distinguish them from non-polaritonic spatial signal oscillations that, for example, are caused by spatial variations of dielectric material properties or by laser intensity fluctuations. Using dispersion calculations based on various optical conductivity models, we found that the polaritons can be explained by simultaneous coupling of THz radiation to various combination of Dirac carriers, massive 2DEG carriers, massive bulk carriers and optical phonons. During the revision of our manuscript, another THz s-SNOM study of polaritons in TIs was published^55^, reporting that massive 2DEGs need to be considered when interpreting THz polaritons in Bi_2_Se_3_. We note, however, that the contribution of massive carriers may be strongly reduced or even absent^2,13^ by growing the Bi_2_Se_3_ by alternative methods, for example by using a buffer layer technique^52,56^. Beyond s-SNOM-based dispersion analysis as demonstrated here, our work paves the way for studying THz polaritons on other TI materials, 2D materials or 2DEGs, such as the mapping of modal field patterns in resonator structures^30^ and moiré superlattices^57^, or the directional propagation on in-plane anisotropic natural materials^42^ and metasurfaces^58^.

## Methods

### Sample preparation

Films of Bi_2_Se_3_ are grown via molecular beam epitaxy (Veeco GenXplor MBE system) on single-side polished sapphire substrate (0001) plane (10 mm × 10 mm × 0.5 mm, MTI Corp., U.S.A.). All films are grown at the same substrate temperature as measured by a non-contact thermocouple (325 °C), growth rate (0.8 nm/min), and selenium: bismuth flux ratio as measured by an ion gauge (≈50), and the selenium cell has a high-temperature cracker zone set at 900 °C^52^. Film thicknesses are determined via x-ray reflection (XRR) measurement. X-ray diffraction further confirmed that only a single phase and one orientation (0001) of Bi_2_Se_3_ has been epitaxially grown on c-direction on the substrate. The sheet concentration (~3.0 × 10^13^ cm^−2^) for 120-nm-thick Bi_2_Se_3_ are obtained via Hall effect measurement in a van der Pauw configuration at room temperature (see Supplementary Note 5), with error bars ~6%.

## Supplementary information

Supplementary Information

## Data Availability

Data that support the results of this work are available upon reasonable request from the corresponding author.
