# Supplementary material for: Real-space nanoimaging of THz polaritons in the topological insulator Bi2Se3
Source: Nat Commun. 2022 Mar 16;13:1374. doi: 10.1038/s41467-022-28791-x (PMC8927118; doi:10.1038/s41467-022-28791-x)
Supplement: Supplementary file 1 — Supplementary Information [file 41467_2022_28791_MOESM1_ESM.pdf]

Supplementary Information for:

Real-space nanoimaging of THz polaritons in the topological insulator Bi_2Se_3

Shu Chen[1], Andrei Bylinkin[1,2], Zhengtianye Wang[3], Martin Schnell[1,4], Greeshma Chandan[3], Peining Li[5], Alexey Y. Nikitin[2,4], Stephanie Law[3], and Rainer Hillenbrand *[,4,6]

[1] *CIC nanoGUNE BRTA, 20018 Donostia - San Sebastián, Spain*

[2] *Donostia International Physics Center (DIPC), 20018 Donostia - San Sebastián, Spain*

[3] *Department of Materials Science and Engineering, University of Delaware, Newark, Delaware, 19716 USA*

[4] *IKERBASQUE, Basque Foundation for Science, 48009 Bilbao, Spain*

[5] *Wuhan National Laboratory for Optoelectronics & School of Optical and Electronic Information, Huazhong University of Science and Technology, Wuhan 430074, China*

[6] *CIC nanoGUNE BRTA and Department of Electricity and Electronics, UPV/EHU, 20018 Donostia-San Sebastián, Spain*

Corresponding author: *r.hillenbrand@nanogune.eu

Supplementary Note 1. THz s-SNOM setup

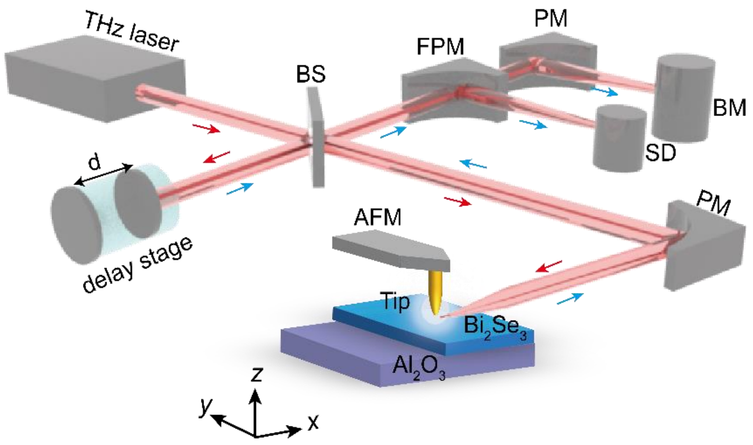

Supplementary Figure 1. THz s-SNOM setup for imaging polaritons in Bi_2Se_3 films. Schematic of THz s-SNOM setup equipped with detectors of bolometer (BM) and Schottky diode (SD). AFM, FPM, PM and BS are atomic force microscopy cantilever, parabolic mirror that can be flipped up and down, parabolic mirror and beam splitter (double-sided polished silicon wafer), respectively.

Supplementary Note 2. Fitting of all near-field line profiles in the complex plane

In Supplementary Figure 2 and 3 we show the data and analysis of all experimental s-SNOM line profiles reported in this work. Analysis follows the procedure demonstrated in Figure 2 of the main text.

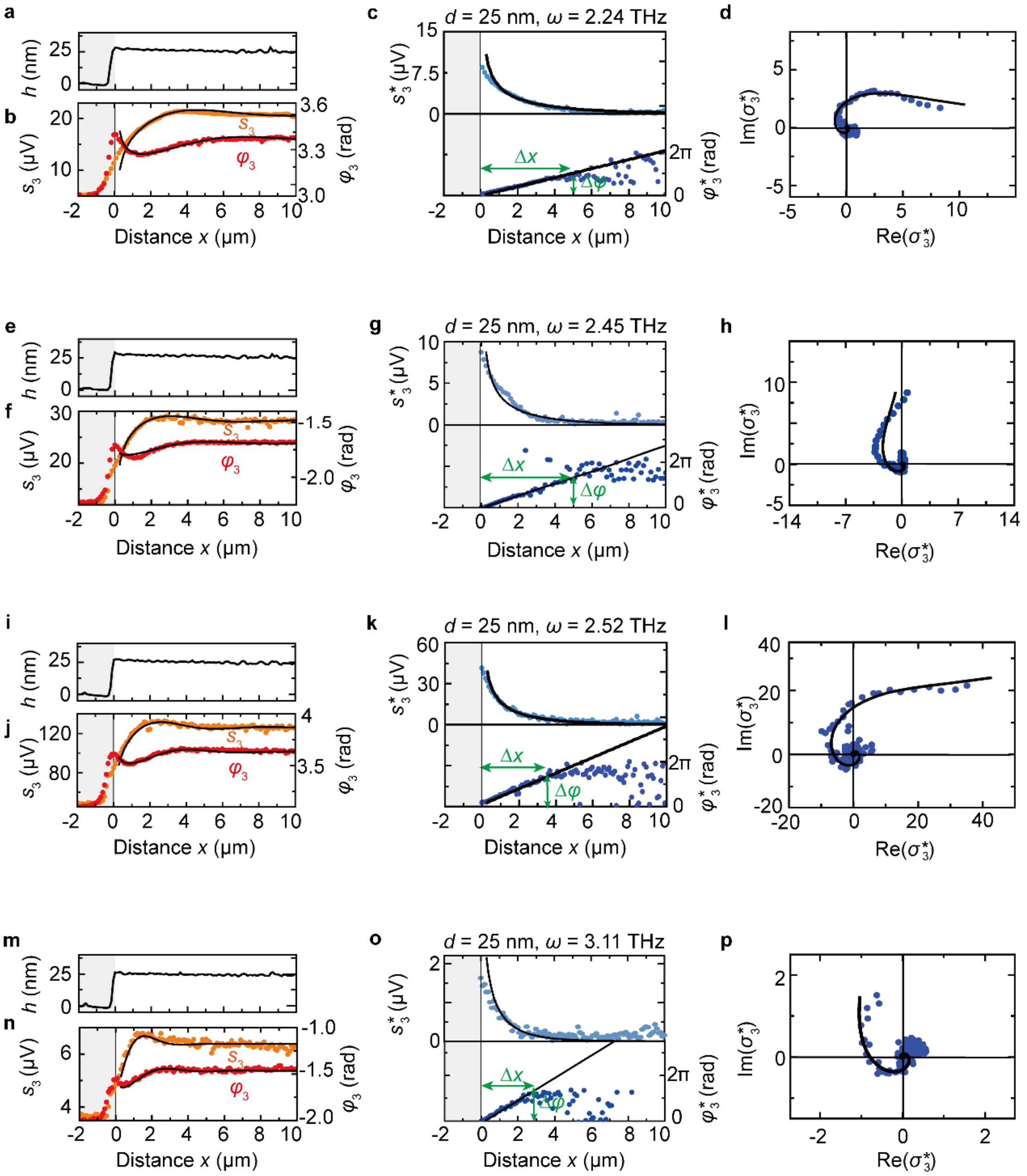

Supplementary Figure 2. Complex-valued analysis of THz near-field line profiles of a 25 nm thick Bi_2Se_3 film. a,e,i,m Topography line profile, showing the height h as measured by AFM. **b,f,j,n** Experimental s-SNOM amplitude and phase line profiles. **c,g,k,o** Amplitude and phase line profiles obtained from the data shown in panels **b,f,j,n** after subtraction of the complex-valued signal offset C at large distances x. **d,h,l,p** Representation of near-field line profiles in the complex plane, after offset subtraction. The black solid lines show the fitting of the experimental data by a radially and exponentially decaying wave, $Ae^{i2k_\mathrm{p}x}/\sqrt{2x}+C$, where C is a constant complex-valued offset.

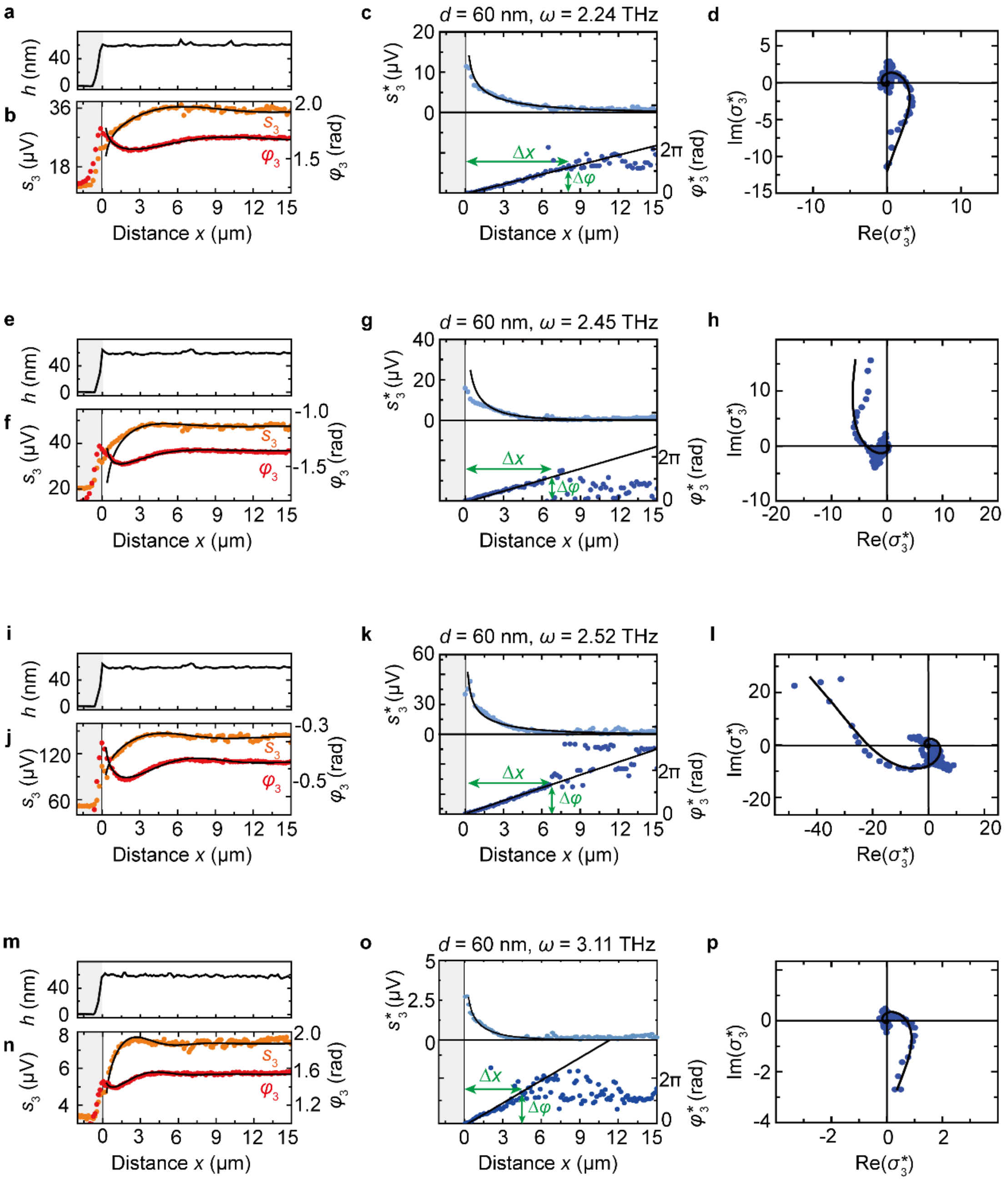

Supplementary Figure 3. Complex-valued analysis of THz near-field line profiles of a 60 nm thick Bi_2Se_3 film. a,e,i,m Topography line profile, showing the height h as measured by AFM. **b,f,j,n** Experimental s-SNOM amplitude and phase line profiles. **c,g,k,o** Amplitude and phase line profiles obtained from the data shown in panels **b,f,j,n** after subtraction of the complex-valued signal offset *C* at large distances x. **d,h,l,p** Representation of near-field line profiles in the complex plane, after offset subtraction. The black solid lines show the fitting of the experimental data by a radially and exponentially decaying wave, $Ae^{i2k_{\mathrm{p}}x}/\sqrt{2x}+C$, where *C* is a constant complex-valued offset.

Supplementary Note 3. Analytical equation for the dispersion of polaritons in a thin topological insulator (TI)

For calculating the polariton dispersions in the main text, we modelled the TI by two conductive layers separated by a dielectric slab of thickness d (see Supplementary Figure 4). The top and bottom layers are modelled as a two-dimensional conductive sheet of a zero thickness with conductivity σ_{Dirac}. The dielectric slab between the two conductive sheets is described by its bulk dielectric function, $\varepsilon_{\mathrm{bulk}}$ (see Supplementary Note 4A). Superstrate (air) and substrate (Al_2O_3) are described by the dielectric constant $\varepsilon_{\mathrm{air}} = 1$ and $\varepsilon_{\mathrm{sub}} = 10$, respectively.

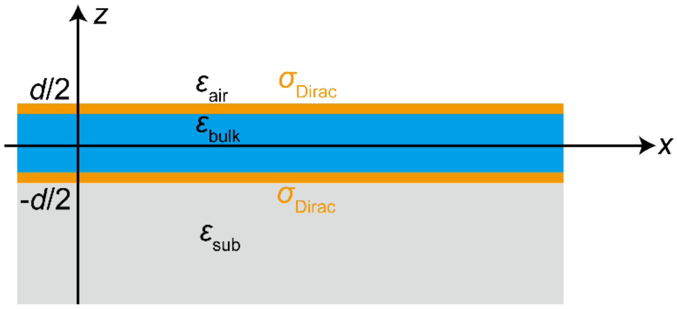

Supplementary Figure 4. Schematic illustration of the TI model

The polariton dispersion relation can be derived by matching the electric and magnetic fields (represented in the form of plane waves) on the TI surfaces using the boundary conditions (see for example Ref.[1]). As a result, we obtain

$$e^{-ik_{\mathrm{bulk,z}}d}\left(\frac{4\pi\sigma_{\mathrm{Dirac}}}{c}+\frac{\varepsilon_{\mathrm{air}}}{q_{\mathrm{air,z}}}+\frac{\varepsilon_{\mathrm{bulk}}}{q_{\mathrm{bulk,z}}}\right)\left(\frac{4\pi\sigma_{\mathrm{Dirac}}}{c}+\frac{\varepsilon_{\mathrm{sub}}}{q_{\mathrm{sub,z}}}+\frac{\varepsilon_{\mathrm{bulk}}}{q_{\mathrm{bulk,z}}}\right)$$
$$= e^{ik_{\mathrm{bulk,z}}d}\left(\frac{4\pi\sigma_{\mathrm{Dirac}}}{c}+\frac{\varepsilon_{\mathrm{air}}}{q_{\mathrm{air,z}}}-\frac{\varepsilon_{\mathrm{bulk}}}{q_{\mathrm{bulk,z}}}\right)\left(\frac{4\pi\sigma_{\mathrm{Dirac}}}{c}+\frac{\varepsilon_{\mathrm{sub}}}{q_{\mathrm{sub,z}}}-\frac{\varepsilon_{\mathrm{bulk}}}{q_{\mathrm{bulk,z}}}\right), \quad (1)$$

where $k_0 = \omega/c$ is the free-space wavevector, k_{p} is the in-plane wavevector, $k_{j,\mathrm{z}} = \sqrt{\varepsilon_j\omega^2/c^2 - {k_{\mathrm{p}}}^2}$, $j = \{\mathrm{air, bulk, sub}\}$ are the out-of-plane wavevectors in the superstrate, slab, and substrate, respectively. $q_{j,\mathrm{z}} = k_{j,\mathrm{z}}/k_0$ and $q = k_{\mathrm{p}}/k_0$ are the normalized wavevectors. Considering large in-plane polariton momenta, we can approximate the out-of-plane wavevector by $k_{j,\mathrm{z}} = \sqrt{\varepsilon_j\omega^2/c^2 - {k_{\mathrm{p}}}^2} \approx ik_{\mathrm{p}}$ and transform Supplementary Equation (1):

$$e^{k_p d}\left(\frac{4\pi\sigma_{\mathrm{Dirac}}}{c}-\frac{\varepsilon_{\mathrm{air}}+\varepsilon_{\mathrm{bulk}}}{q}i\right)\left(\frac{4\pi\sigma_{\mathrm{Dirac}}}{c}-\frac{\varepsilon_{\mathrm{sub}}+\varepsilon_{\mathrm{bulk}}}{q}i\right)$$
$$= e^{-k_p d}\left(\frac{4\pi\sigma_{\mathrm{Dirac}}}{c}-\frac{\varepsilon_{\mathrm{air}}-\varepsilon_{\mathrm{bulk}}}{q}i\right)\left(\frac{4\pi\sigma_{\mathrm{Dirac}}}{c}-\frac{\varepsilon_{\mathrm{sub}}-\varepsilon_{\mathrm{bulk}}}{q}i\right). \quad (2)$$

If we consider a symmetric dielectric environment of the TI, i.e. setting $\varepsilon_{\text{air}} = \varepsilon_{\text{sub}} = \varepsilon$, Supplementary Equation (2) splits into the two independent dispersions for two modes:

$$e^{k_{\text{p}}d}\left(\frac{4\pi\sigma_{\text{Dirac}}}{c} - \frac{\varepsilon+\varepsilon_{\text{bulk}}}{q}i\right) \pm \left(\frac{4\pi\sigma_{\text{Dirac}}}{c} - \frac{\varepsilon-\varepsilon_{\text{bulk}}}{q}i\right) = 0. \quad (3)$$

The sign " + " corresponds to an optical (symmetric) mode, with the charges oscillating in-phase in both conductive layers, while " − " corresponds to an acoustic (antisymmetric) mode with the charges oscillating out-of-phase and electric field confined inside the slab.

In the general case of the non-symmetric dielectric surrounding we can assume that the TI layer thickness is much smaller than the polariton wavelength ($k_{\text{p}}d \ll 1$). In this case we can expand the exponentials $e^{\pm k_{\text{p}}d}$ into a Taylor series in $k_{\text{p}}d$ and retain the first nonvanishing terms, $e^{\pm k_{\text{p}}d} = 1 \pm k_{\text{p}}d$. Additionally, taking into account that $\varepsilon_{\text{air,sub}} \ll \varepsilon_{\text{bulk}}$, we can significantly simplify Supplementary Equation (2) for the optical mode to[2]

$$q = \frac{k_{\text{p}}}{k_0} = i\frac{c}{4\pi}\frac{\varepsilon_{\text{sub}}+\varepsilon_{\text{air}}}{\sigma_{\text{bulk}}+2\sigma_{\text{Dirac}}} = i\frac{c}{4\pi}\frac{\varepsilon_{\text{sub}}+\varepsilon_{\text{air}}}{\sigma}, \quad (4)$$

where $\sigma_{\text{bulk}} = \frac{\omega d \varepsilon_{\text{bulk}}}{4\pi i}$ is the effective 2D conductivity of the slab (bulk) and σ is the total effective 2D conductivity of the TI[3-5].

Supplementary Equation (4) can also be easily obtained using Ohm's law for parallel-connected 2D conductive elements (two elements with σ_{Dirac} and one element with σ_{bulk}). Moreover, Supplementary Equation (4) can be generalized to account for a massive two-dimensional electron gas (2DEG) on the top and bottom interfaces of the TI, which could exist due to surface band bending[6-9]. In the presence of a massive 2DEG, the total effective conductivity of TI is given by $\sigma = \sigma_{\text{bulk}} + 2\sigma_{\text{Dirac}} + 2\sigma_{\text{2DEG}}$, assuming that the surface carriers are the same on top and bottom interfaces. Importantly, Supplementary Equation (4) yields the same results as the equation for calculating the polariton dispersions in Bi_2Se_3 that are reported in literature [2, 7, 8, 10].

The assumption $\varepsilon_{\text{air,sub}} \ll |\varepsilon_{\text{bulk}}|$ is clearly fulfilled in our experiments, which can be seen in Supplementary Figure 5a, where we show $\varepsilon_{\text{bulk}}$, which takes into account the massive bulk carriers and phonons with bulk carrier concentration of n_{bulk}= 3.72·10^{18} cm^{-3} (Supplementary Equations (5) to (7) and Table 1). The comparison between the analytically and numerically calculated polariton dispersion (Supplementary Figure 5b) indeed shows good agreement. We repeated the calculations for $\varepsilon_{\text{bulk}} = 22$ (close to the high-frequency permittivity of Bi_2Se_3 from the contribution of the bandgap, ε_{bg}, see Supplementary Equation (5), i.e., in absence of bulk carriers and phonons) and find that the analytical solution is still valid.

We note that the numerical simulation shown in Supplementary Figure 5 fully considers the

anisotropic bulk permittivity of Bi_2Se_3 (i.e. anisotropic optical phonons and bulk plasmons), whereas the analytical simulation considers only the in-plane bulk permittivity. The excellent agreement between numerical and analytical calculation verifies the validity of the 2D conductivity sheet model. Particularly, it shows that the anisotropic bulk properties (and related hyperbolic polariton dispersion) do not need to be considered for analyzing the polariton dispersion.

We also note that Bi_2Se_3 is a quite lossy material, which can be concluded from the very short polariton propagation lengths. Higher order hyperbolic polariton modes (which in principle can exist in uniaxial materials such as Bi_2Se_3) are thus strongly damped and can be neglected, as pointed out in Refs[5,11].

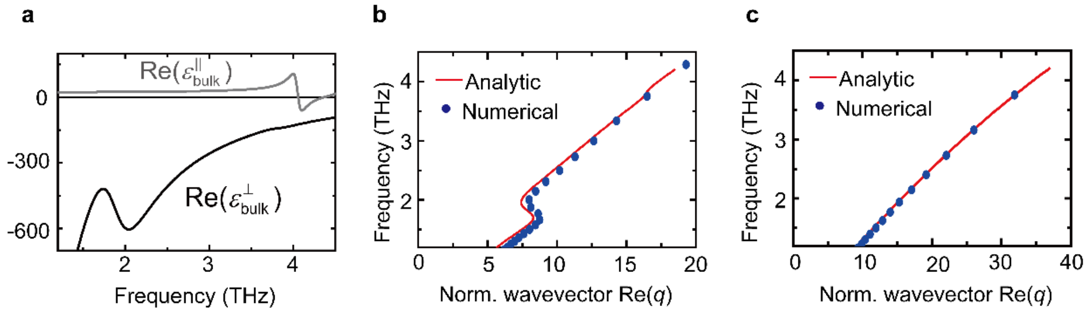

Supplementary Figure 5. Comparison of analytical and numerical dispersion calculations. a Anisotropic bulk permittivity $\varepsilon_{\mathrm{bulk}}$ of the Bi_2Se_3 layers of our experiments. For the in-plane permittivity we consider the in-plane the optical phonons and the in-plane conductivity due to massive carriers (Drude term) according to $\varepsilon_{\mathrm{bulk}}^{\perp} = \varepsilon_{\mathrm{phonon}} + \varepsilon_{\mathrm{Drude}}$ described by Supplementary Equations (5)-(7) and Table 1. For the bulk carrier concentration we use $n_{\mathrm{bulk}} = 3.72 \cdot 10^{18}$ cm^{-3}. For the out-of-plane permittivity we us $\varepsilon_{\mathrm{bulk}}^{\parallel} = 17.4 + \frac{\omega_{\mathrm{p},1}^2}{\omega_{0,1}^2 - \omega^2 - i\omega\gamma_1} + \frac{\omega_{\mathrm{p},2}^2}{\omega_{0,2}^2 - \omega^2 - i\omega\gamma_2}$ with $\omega_{\mathrm{p},1} = 283$ cm^{-1}, $\omega_{0,1} =$ 135 cm^{-1}, $\omega_{\mathrm{p},2} = 156$ cm^{-1}, $\omega_{0,2} = 154$ cm^{-1}, $\gamma_1 = \gamma_2 = 3.5$ cm^{-1} (Ref. 12). Black and grey curves show the real part of $\varepsilon_{\mathrm{bulk}}^{\perp}$and $\varepsilon_{\mathrm{bulk}}^{\parallel}$, respectively. **b** Comparison of analytically (solid line, obtained with Supplementary Equation (4)) and numerically (blue symbols, obtained from COMSOL mode solver) calculated polariton dispersions for a 25 nm thick Bi_2Se_3 layer. In the numerical simulation we consider a 25 nm thick slab with the anisotropic bulk permittivity $\varepsilon_{\mathrm{bulk}}$ of panel a and a bottom and a top conduction layer with a carrier concentration of $n_{\mathrm{Dirac}} = 1.25 \cdot 10^{13}$ cm^{-2}. In the analytical calculation, only the in-plane permittivity $\varepsilon_{\mathrm{bulk}}^{\perp}$ of panel a is considered. **c** Comparison of analytically and numerically calculated polariton dispersion for an isotropic bulk permittivity $\varepsilon_{\mathrm{bulk}} = 22$ with a bottom and top conduction layer of a carrier concentration of $n_{\mathrm{Dirac}} = 1.25 \cdot 10^{13}$ cm^{-2}.

Supplementary Note 4. Bulk dielectric function and surface conductivity of Bi_2Se_3

A. Bulk dielectric function of Bi_2Se_3

Due to its layered structure, Bi_2Se_3 is a uniaxial material. Consequently, its dielectric function, $\varepsilon_{\text{bulk}}$, has to be described by a uniaxial tensor, where the in-plane components (parallel to the surface and being the same) are different to the out-of-plane component[12]. However, for describing polaritons in layers that are much thinner than the polariton wavelength, the out-of-plane component can be neglected (for verification see Supplementary Figure 5), apart from its sign, which determines the sign of the phase velocity of the polaritons and depends on the sign of the in-plane components[3-5]. This condition is fulfilled in our experiments, as the Bi_2Se_3 thickness is not more than $d =$ 60 nm and the polariton wavelengths are larger than several micrometer (Supplementary Figure 5). We thus describe $\varepsilon_{\text{bulk}}$ as a scalar using the in-plane permittivity components.

In the considered frequency range, the-in plane dielectric function of Bi_2Se_3 is governed by in-plane optical phonons ($\varepsilon_{\text{phonon}}$) and plasmons ($\varepsilon_{\text{Drude}}$) due to unavoidable bulk carriers[8,10,13,14]. We describe it by

$$\varepsilon_{\text{bulk}} = \varepsilon_{\text{phonon}} + \varepsilon_{\text{Drude}} \tag{5}$$

With

$$\varepsilon_{\text{phonon}} = \varepsilon_\infty + \varepsilon_{\text{bg}} + \varepsilon_\alpha + \varepsilon_\beta = \varepsilon_\infty + \frac{\omega_{\text{p,bg}}^2}{\omega_{0,\text{bg}}^2 - \omega^2 - i\omega\gamma_{\text{bg}}} + \frac{\omega_{\text{p},\alpha}^2}{\omega_{0,\alpha}^2 - \omega^2 - i\omega\gamma_\alpha} + \frac{\omega_{\text{p},\beta}^2}{\omega_{0,\beta}^2 - \omega^2 - i\omega\gamma_\beta} \tag{6}$$

and

$$\varepsilon_{\text{Drude}} = -\frac{\omega_{\text{p,D}}^2}{\omega^2 + i\omega\gamma_{\text{D}}} \tag{7}$$

where $\varepsilon_\infty = 1$, ε_{bg}, ε_α and ε_β represent the high-frequency permittivity, contribution of the bandgap and the two in-plane optical phonons, respectively[8, 10, 13, 14]. The oscillator strengths $\omega_{\text{p},x}$, oscillator frequencies $\omega_{0,x}$ and damping parameters γ_x ($x = \text{D}, \text{bg}, \alpha$ and β) are summarized in Supplementary Table 1.

	$\omega_{\text{p},x}$ [cm^{-1}]	$\omega_{0,x}$ [cm^{-1}]	γ_x [cm^{-1}]
$\varepsilon_{\text{Drude}}$	$\omega_{\text{p,D}}$	0	7.43
ε_{bg}	11249	2029.5	3920.5
ε_α	675.9	63.03	17.5
ε_β	100	126.94	10

Supplementary Table 1. Parameters for the bulk dielectric function of Bi_2Se_3.

The plasma frequency is given by $\omega_{\text{p,D}} = \sqrt{\frac{4\pi n_{\text{bulk}} e^2}{m^*}}$, where $m^* = 0.15 m_e$ is the effective electron mass, m_e the electron mass and n_{bulk} the bulk free carrier concentration.

B. Surface conductivity of Dirac carries

The surface conductivity of the Bi_2Se_3 films due to Dirac carriers (surface states) is described by[12]

$$\sigma_{\mathrm{Dirac}} = \frac{e^2 kT \ln\left[2\cosh\left(\frac{E_{\mathrm{F}}}{2kT}\right)\right]}{2\hbar^2\pi} \frac{i}{\omega + i\gamma_{\mathrm{Dirac}}} \quad (8)$$

where γ_{Dirac} and T are carrier relaxation rate and temperature, respectively. The Fermi energy for a single surface is given by $E_{\mathrm{F}} = \hbar v_{\mathrm{F}}\sqrt{4\pi n_{\mathrm{Dirac}}}$, where $v_{\mathrm{F}} = 5\cdot10^5$ m/s is the Fermi velocity[15, 16]. We assume that $\gamma_{\mathrm{Dirac}} = 0$ and note that variation of this value does not significantly influence the polariton dispersion. The interband conductivity is ignored, as it is negligible at THz frequencies for the considered Fermi energies ($2E_{\mathrm{F}} > \hbar\omega$). For the temperature we use $kT = 25$ meV (300 K).

We note that at low temperatures or large Fermi energies, $E_{\mathrm{F}} \gg kT$, the Dirac conductivity is of the form $\sigma_{\mathrm{Dirac}} = \frac{e^2 E_{\mathrm{F}}}{4\hbar^2\pi} \frac{i}{\omega + i\gamma_{\mathrm{Dirac}}}$.

C. Surface conductivity of a 2DEG formed by massive carriers

The surface conductivity of a 2DEG formed by massive carriers (extremely thin layers that can be regarding as 2D sheets of zero thickness[2,7]) in Bi_2Se_3 is modelled within the local approximation by[17]

$$\sigma_{\mathrm{2DEG}} = \frac{e^2 n_{\mathrm{2DEG}}}{m^*} \frac{i}{\omega + i\gamma_{\mathrm{2DEG}}}, \quad (9)$$

where n_{2DEG}, $m^* = 0.15 m_e$ and γ_{2DEG} are the carrier concentration, effective mass and carrier relaxation rate, respectively. We assume that $\gamma_{\mathrm{2DEG}} = 0$ and note that variation of this value does not significantly influence the polariton dispersion.

D. Carrier concentrations used in Figure 4 of the main text

The bulk carrier concentration (n_{bulk}), Dirac carrier concentration (n_{Dirac}) and 2DEG carrier concentration (n_{2DEG}) used for calculating the dispersion curves shown in Figure 4b-d of the main text are listed on the right side of Supplementary Figure 6.

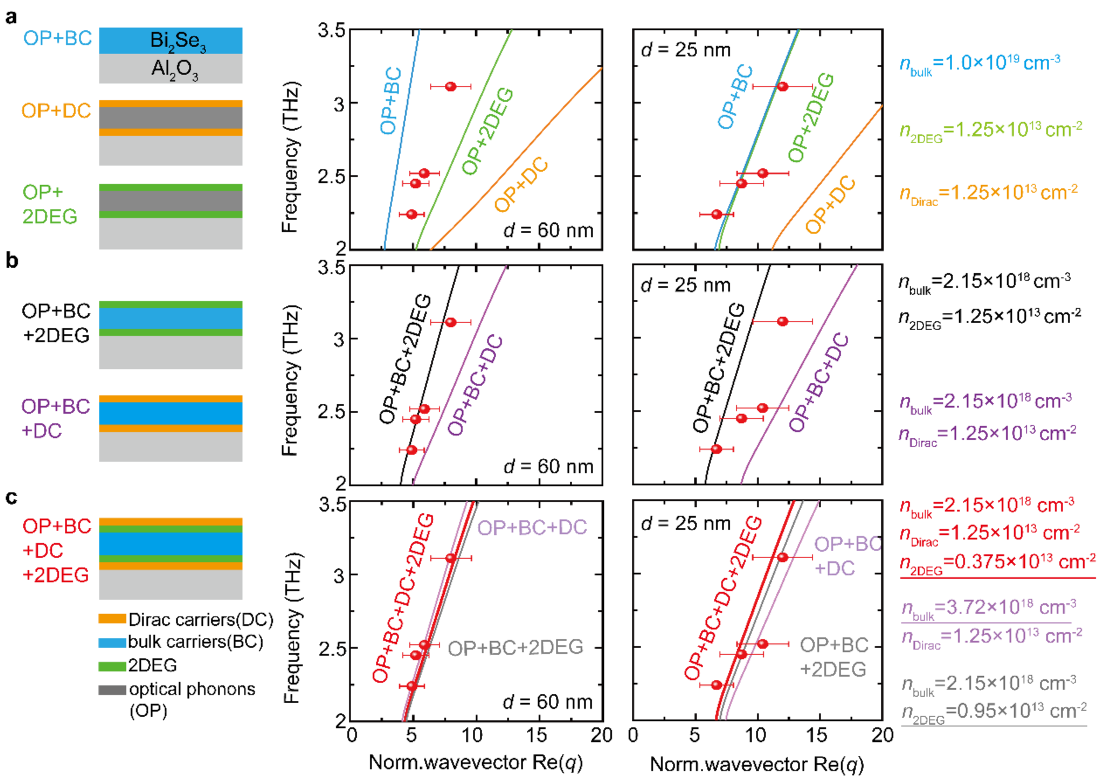

Supplementary Figure 6. Polariton dispersions in Bi_2Se_3 films with 60 nm and 25 nm thickness. **a-c** Red symbols in the diagrams show the polariton dispersions obtained by complex-valued fitting of experimental line profiles as demonstrated in Figure 2 of the main text. Error bars indicate a 20% uncertainty of the wavevector, which we estimate conservatively from comparison of experimental and simulated near-field line profiles in the main text. Solid lines show calculated dispersions based on various conductivity models (described in main text), which are sketched on the left side. We consider various optical conductivity contributions based on optical bulk phonons (OP), massive bulk carriers (BC), Dirac carriers (DC) at both Bi_2Se_3 surfaces, and massive two-dimensional electron gases (2DEG) at both Bi_2Se_3 surfaces. The carrier concentrations of bulk carriers, Dirac carriers and 2DEG used for calculating each dispersion curve are correspondingly shown on the right side. Fit parameters are underlined.

Supplementary Note 5. Resistance and Hall resistance measurements at room temperature

Bi_2Se_3 films were grown on 10 mm by 10 mm large sapphire substrates of 0.5 mm thickness. The resistance and Hall resistance measurements were done immediately after taking the samples out of growth chamber. To that end, Indium contacts were soldered at the four corners of the sample (as shown in Supplementary Figure 7a) and contacted with copper probes for the resistance and Hall resistance measurements via Van der Pauw method[18]. The contacts were labeled from 1 to 4 in counterclockwise direction. We defined the current (I_{12}, I_{23}, I_{13}, I_{24}), voltage (V_{14}, V_{43}, V_{13}, V_{24}) as follows:

I_{12}: current from contact 1 to contact 2
I_{23}: current from contact 2 to contact 3
I_{13}: current from contact 1 to contact 3
I_{24}: current from contact 2 to contact 4
V_{14}: Voltage between contact 1 and 4, $V_1 - V_4$
V_{43}: Voltage between contact 4 and 3, $V_4 - V_3$
V_{13}: Voltage between contact 1 and 3, $V_1 - V_3$
V_{24}: Voltage between contact 2 and 4, $V_2 - V_4$

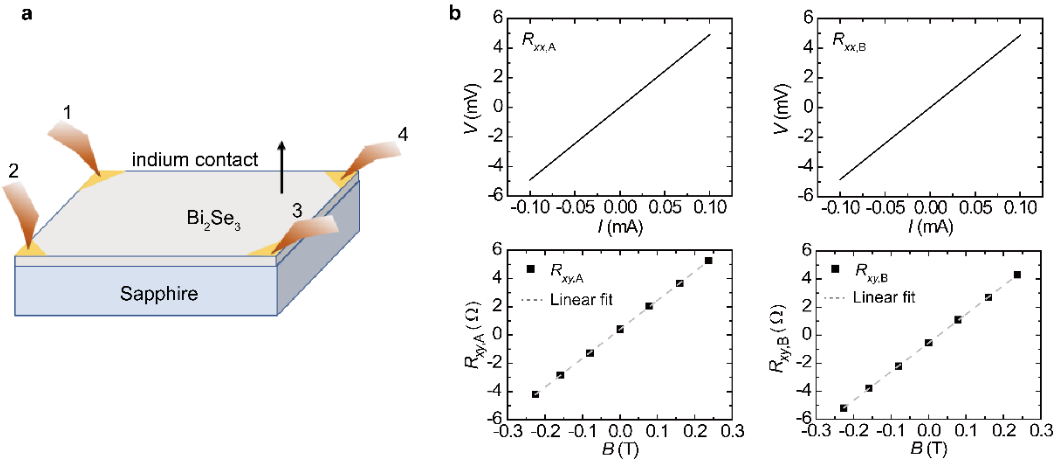

Supplementary Figure 7. Resistance and Hall resistance measurements. a Schematic of the van der Pauw measurement configuration. **b** Top: Voltage as function of current, $V(I)$, for measurement of longitudinal resistances $R_{xx,A}$ and $R_{xx,B}$. Bottom: Hall resistance (black symbols) as a function of magnetic field, $R_{xy}(B)$. In the Hall measurement, a constant current of 0.1 mA was used while the magnetic field was swept from -0.2 T to 0.2 T. From the measured Hall voltages, we obtained the resistances $R_{xy,A}$ and $R_{xy,B}$ according to Supplementary Equations (14) and (15). The grey dashed lines show linear fits of the experimental data. The Bi_2Se_3 film thickness is 120 nm.

In the resistance measurement, we considered two cases. Case A: we measured the current from contact 1 to 2 and voltage between contact 4 and 3. Case B: we measured the current from contact 2 to 3 and voltage between contact 1 and 4. The longitudinal resistance ($R_{xx,\mathrm{A}}, R_{xx,\mathrm{B}}$) of both cases are defined as:

$$R_{xx,\mathrm{A}} = V_{43}/I_{12} \tag{10}$$
$$R_{xx,\mathrm{B}} = V_{14}/I_{23} \tag{11}$$

We obtained $R_{xx,\mathrm{A}}$ =48.99 Ω, $R_{xx,\mathrm{B}}$ =48.52 Ω from the slopes of the $V(I)$ curves in the top panel of Supplementary Figure 7b. We thus obtained the sheet resistance R_s = 220.98 Ω using the Van der Pauw equation:

$$e^{(-\pi R_{xx,\mathrm{A}}/R_\mathrm{S})} + e^{(-\pi R_{xx,\mathrm{B}}/R_\mathrm{S})} = 1. \tag{12}$$

The resistivity ρ = 0.0022 cm^{-1} was obtained from

$$\rho = R_s d, \tag{13}$$

where d is the thickness of Bi_2Se_3 film.

For the Hall resistance measurements, a magnetic field B perpendicular to the film was applied. The current and voltage probes were placed diagonally. We also considered two cases. Case A: we measured the current from contact 1 and 3 and the voltage between contact 2 and 4. Case B: we measured the current from contact 2 and 4 and the voltage between contact 1 and 3. The Hall resistance of both cases are given by:

$$R_{xy,\mathrm{A}} = V_{24}/I_{13} \tag{14}$$

$$R_{xy,\mathrm{B}} = V_{13}/I_{24}. \tag{15}$$

During the Hall measurement, we used a constant current of 0.1 mA and swept the magnetic field from -0.2 to 0.2 T. The bottom panel of Supplementary Figure 7b shows the Hall resistances $R_{xy,\mathrm{A}}$ and $R_{xy,\mathrm{B}}$ as a function of B, revealing that the Hall resistance is linear to the magnetic field. The sheet carrier concentrations $n_{s,\mathrm{A}}$ and $n_{s,\mathrm{B}}$ obtained from both measurements were obtained according to

$$n_{\mathrm{s,A}} = 1/(e \cdot (R_{xy,\mathrm{A}}/B)) \tag{16}$$

$$n_{\mathrm{s,B}} = 1/(e \cdot (R_{xy,\mathrm{B}}/B)) \tag{17}$$

$$n_{\mathrm{s}} = \frac{n_{\mathrm{s,A}} + n_{\mathrm{s,B}}}{2}. \tag{18}$$

We obtained $n_{\mathrm{s,A}}$= 3.05·10^{13} cm^{-2}, $n_{\mathrm{s,B}}$= 3.04·10^{13} cm^{-2} and the average value n_{s} = 3.04·10^{13} cm^{-2}. The carrier mobility μ was obtained according to $\mu = 1/(e \cdot n_{\mathrm{s}} \cdot R_{\mathrm{s}})$ = 927 cm^2/(V·s). We repeated the above-described Hall measurements three times and obtained averaged values n_{s} = 3.0·10^{13} cm^{-2} and μ = 925.6 cm^2/(V·s).

Supplementary Note 6. Hall effect of films with multi-conduction channels

If there are multiple conduction channels in a thin film (one conduction channel corresponds to one type of carries), no matter what types of carriers are measured, the general longitudinal conductivity σ_{xx} and transverse conductivity σ_{xy} (also called Hall conductivity) can be expressed as:

$$\sigma_{xx}(B) = \sum_{j=1}^{N} \sigma_{xx,j} = \sum_{j=1}^{N} \frac{q_j n_j \mu_j}{1+(\mu_j B)^2} \tag{19}$$

$$\sigma_{xy}(B) = \sum_{j=1}^{N} \sigma_{xy,j} = B \sum_{j=1}^{N} \mu_j \sigma_{xx,j} = \sum_{j=1}^{N} \frac{q_j n_j {\mu_j}^2 B}{1+(\mu_j B)^2}. \tag{20}$$

N is the number of total conduction channels, $\sigma_{xx,j}$ and $\sigma_{xy,j}$ are the longitudinal

conductivity transverse conductivity of the j_{th} channel, respectively. The parameters q_j, n_j and μ_j are the charge, carrier density and mobility of the j_{th} channel, respectively. The resistivity ρ and Hall coefficient R_{H} of the whole system can be expressed as:

$$\rho = \frac{\sigma_{xx}}{{\sigma_{xx}}^2 + {\sigma_{xy}}^2} \tag{21}$$

$$R_{\mathrm{H}} = \frac{\sigma_{xy}}{B \cdot ({\sigma_{xx}}^2 + {\sigma_{xy}}^2)}. \tag{22}$$

R_{H} is related with Hall resistance R_{xy} as

$$R_{xy} = R_{\mathrm{H}} B. \tag{23}$$

The overall mobility μ and sheet carrier concentration n_{s} are obtained according to:

$$\mu = R_{\mathrm{H}} / \rho \tag{24}$$

$$n_{\mathrm{s}} = 1/(q \cdot R_{\mathrm{H}})\,. \tag{25}$$

For two conduction channels, we have a general form of μ, n_{s} at any magnetic field B:

$$\mu = \frac{n_2 {\mu_2}^2 (1 + {\mu_1}^2 B^2) + n_1 {\mu_1}^2 (1 + {\mu_2}^2 B^2)}{n_2 \mu_2 (1 + {\mu_1}^2 B^2) + n_1 \mu_1 (1 + {\mu_2}^2 B^2)} \tag{26}$$

$$n_{\mathrm{s}} = \frac{{n_2}^2 {\mu_2}^2 (1 + {\mu_1}^2 B^2) + {n_1}^2 {\mu_1}^2 (1 + {\mu_2}^2 B^2) + 2 n_1 n_2 \mu_1 \mu_2 (1 + \mu_1 \mu_2 B^2)}{n_1 {\mu_1}^2 (1 + {\mu_2}^2 B^2) + n_2 {\mu_2}^2 (1 + {\mu_1}^2 B^2)} \tag{27}$$

The bottom panel of Supplementary Figure 7b shows that R_{xy} is linearly changing with B, indicating that R_{H} is a constant value according to Supplementary Equation (23). We conclude that n_{s} and μ are independent on B. In the following we thus discuss two special cases when n_{s} and μ are independent on B.

(a) When $\mu_1 = \mu_2$ we obtain according to Supplementary equations (26) and (27):

$$\mu = \mu_1 = \mu_2 \tag{28}$$

$$n_{\mathrm{s}} = n_1 + n_2, \tag{29}$$

which implies that the total sheet carrier concentration is simply the sum of the individual concentrations n_1 and n_2 of the two types of carriers, provided that they have the same mobility. From the pure Hall measurement, we cannot judge whether this is the case of our sample. However, far-field THz spectroscopy of Bi_2Se_3 microribbons revealed that the polariton dispersions can be explained only when the sum of the sheet carrier concentration of bulk carriers and Dirac carriers is much larger than the carrier concentration obtained from Hall measurements[8,19]. The same observation is made in the main text of this manuscript. We thus can exclude this case.

(b) When $\mu^2 B^2 << 1$, Supplementary Equations (26) and (27) can be simplified as follows:

$$\mu = \frac{n_1\mu_1{}^2 + n_2\mu_2{}^2}{n_1\mu_1 + n_2\mu_2} \tag{30}$$

$$n_\text{s} = \frac{(n_1\mu_1 + n_2\mu_2)^2}{n_1\mu_1{}^2 + n_2\mu_2{}^2} \tag{31}$$

In our Hall measurements this condition was fulfilled, as the maximum magnetic field was 0.2 T and the carrier mobility is about $\mu \sim 1000$ cm^2/(V·s).

Supplementary Equations (30) and (31) show that n_s of our sample is not just from the contribution of one type of carrier. However, a mere Hall measurement of a single film does not allow us to separate the contributions of two carriers. By performing Hall measurement for differently thick films, we found that n_s exhibits a clear thickness dependence for thicker films (Supplementary Figure 8). For thin films, we find that n_s is around $2.5 \cdot 10^{13}$ cm^{-2} and nearly independent of the film thickness (indicated by the orange horizontal line in Supplementary Figure 8). From the thickness independence we conclude that the measurements of thin films yield and estimate for the carrier concentration at the surfaces of our samples. Indeed, this value of $n_\text{2D,Hall} = 2.5 \cdot 10^{13}$ cm^{-2} is very close to values reported for Dirac carriers in Refs.[20-22]. Assuming further that the mobility of Dirac carriers is larger than that of massive bulk carriers (due to topological protection)[23], we can thus conclude that most of the carriers measured in our Hall measurement can be attributed to Dirac carriers, i.e. Supplementary Equation (31) reduces to $n_\text{s} \approx n_1$ for $n_1 > n_2$ and $\mu_1 > \mu_2$ (indices 1 and 2 representing Dirac and massive bulk carriers, respectively).

On the other hand, the increasing carrier concentration with increasing film thickness (for thicker films) indicates that bulk carriers exist in our Bi_2Se_3 films (marked by Δn_s in Supplementary Figure 8). We estimate the three-dimensional (3D) bulk carrier concentration as $n_\text{bulk} = (\Delta n_\text{s}/170\,nm) = 2.15 \cdot 10^{18}$ cm^{-3} in our Bi_2Se_3 films and assume that it is independent of film thickness[24].

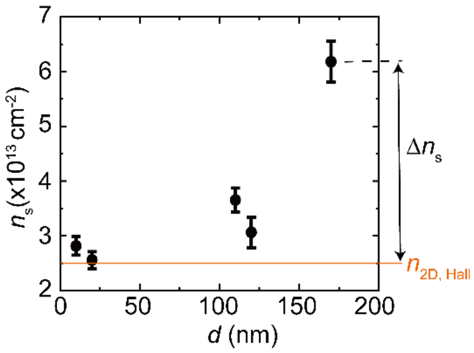

Supplementary Figure 8. Thickness-dependent sheet carrier concentration from Hall measurements. The data were taken from Bi_2Se_3 films that were grown under the same conditions as the Bi_2Se_3 films reported in the main text.

One may argue that $n_{2D,Hall}$ = 2.5·10^{13} cm^{-2} for the surface carriers originates from massive 2DEG carriers. However, the typical massive 2DEG carrier concentrations reported in literature for Bi_2Se_3 films are typically smaller [7,9,20]. Indeed, by assuming that $n_{2D,Hall}$ = 2.5·10^{13} cm^{-2} is exclusively forming a massive 2DEG, the calculated polariton dispersions do not match the experimental polariton dispersions (see green curves in Figure 4b and black curves in Figure 4c of the main text). A more reasonable assumption is that $n_{2D,Hall}$ = 2.5·10^{13} cm^{-2} reveals Dirac carriers and that a small amount of 2DEG carriers co-exist. With such an assumption we can actually well fit the experimental polariton dispersion (see red curves in Figure 4d of the main text).

Generally, separating the contributions of two types of carriers in TIs by Hall measurements may be achieved at low temperatures (~1.5 K) and strong magnetic fields (above 2 T)[20,22]. In that case, the $R_{xy}(B)$ curves are typically non-linear[20,22]. Fitting the nonlinear curve allows to extract the individual sheet concentration and mobility of each type

Supplementary References

1. Svintsov, D., Vyurkov, V., Ryzhii, V. & Otsuji, T. Voltage-controlled surface plasmon-polaritons in double graphene layer structures. *J. Appl. Phys.* **113**, 053701 (2013).
2. Stauber, T., Gómez-Santos, G. & Brey, L. Spin-charge separation of plasmonic excitations in thin topological insulators. *Phys. Rev. B* **88**, 205427 (2013).
3. Gomez-Diaz, J. S., Tymchenko, M. & Alu, A. Hyperbolic plasmons and topological transitions over uniaxial metasurfaces. *Phys. Rev. Lett.* **114**, 233901 (2015).
4. Nikitin, A. Y. Graphene plasmonics. *in World Scientific Handbook of Metamaterials and Plasmonics*, vol 4: Recent progress in the field of nanoplasmonics (ed. Aizpurua, J.) (World Scientific, 2017).
5. Alfaro-Mozaz, F. J. et al. Deeply subwavelength phonon-polaritonic crystal made of a van der Waals material. *Nat. Commun.* **10**, 42 (2019).
6. Bianchi, M., Guan, D., Bao, S., Mi, J., Iversen, B. B., King, P. D. & Hofmann, P. Coexistence of the topological state and a two-dimensional electron gas on the surface of Bi_2Se_3. *Nat. Commun.* **1**, 128 (2010).
7. Sim, S. et al. Ultra-high modulation depth exceeding 2,400% in optically controlled topological surface plasmons. *Nat. Commun.* **6**, 8814 (2015).
8. Ginley, T. P. & Law, S. Coupled Dirac plasmons in topological insulators. *Adv. Opt. Mater.* **6**, 1800113 (2018).
9. Mooshammer, F. et al. Nanoscale near-field tomography of surface states on $(Bi_{0.5}Sb_{0.5})_2Te_3$. *Nano. Lett* **18**, 7515-7523 (2018).
10. Deshko, Y., Krusin-Elbaum, L., Menon, V., Khanikaev, A. & Trevino, J. Surface plasmon polaritons in topological insulator nano-films and superlattices. *Opt. Express* **24**, 7398-7410 (2016).

11. Álvarez-Pérez, G., Voronin, K. V., Volkov, V. S., Alonso-González, P. & Nikitin, A. Y. Analytical approximations for the dispersion of electromagnetic modes in slabs of biaxial crystals. *Phys. Rev. B* **100**, 235408 (2019).

12. Wu, J. S., Basov, D. N. & Fogler, M. M. Topological insulators are tunable waveguides for hyperbolic polaritons. *Phys. Rev. B* **92**, 205430 (2015).

13. Dordevic, S. V., Wolf, M. S., Stojilovic, N., Lei, H. & Petrovic, C. Signatures of charge inhomogeneities in the infrared spectra of topological insulators Bi_2Se_3, Bi_2Te_3 and Sb_2Te_3. *J. Phys. Condens. Matter* **25**, 075501 (2013).

14. Wolf, M. S. Infrared and optical studies of topological insulators Bi_2Te_3, Bi_2Se_3 and Sb_2Te_3. *Thesis*. (The University of Akron, 2011).

15. Zhang, H. J., Liu, C. X., Qi, X. L., Dai, X., Fang, Z. & Zhang, S. C. Topological insulators in Bi_2Se_3, Bi_2Te_3 and Sb_2Te_3 with a single Dirac cone on the surface. *Nature Phys.* **5**, 438-442 (2009).

16. Xie, Z. J. et al. Orbital-selective spin texture and its manipulation in a topological insulator. *Nat. Commun.* **5**, 3382 (2014).

17. Mikhailov, S. A. Plasma instability and amplification of electromagnetic waves in low-dimensional electron systems. *Phys. Rev. B* **58**, 1517-1532 (1998).

18. Van der Pauw, L. J. A method of measuring specific resistivity and Hall effect of discs of arbitrary shapes. *Philips. Res. Repts.* **13**, 1-9 (1958).

19. Wang, Y. & Law, S. Propagating Dirac plasmon polaritons in topological insulators. *J. Opt.* **22**, 125001 (2020).

20. Bansal, N., Kim, Y. S., Brahlek, M., Edrey, E. & Oh, S. Thickness-independent transport channels in topological insulator Bi_2Se_3 thin films. *Phys. Rev. Lett.* **109**, 116804 (2012).

21. Di Pietro, P. et al. Observation of Dirac plasmons in a topological insulator. *Nat. Nanotechnol.* **8**, 556-560 (2013).

22. Brahlek, M., Kim, Y. S., Bansal, N., Edrey, E. & Oh, S. Surface versus bulk state in topological insulator Bi_2Se_3 under environmental disorder. *Appl. Phys. Lett.* **99**, 012109 (2011).

23. Moore, J. E. The birth of topological insulators. *Nature* **464**, 194-198 (2010).

24. Kim, Y. S. et al. Thickness-dependent bulk properties and weak antilocalization effect in topological insulator Bi_2Se_3. *Phys. Rev. B* **84**, 073109 (2011).
